# Assessment of Beliefs and Attitudes About Statins Posted on Twitter: A Qualitative Study

**DOI:** 10.1001/jamanetworkopen.2020.8953

**Published:** 2020-06-25

**Authors:** Su Golder, Karen O’Connor, Sean Hennessy, Robert Gross, Graciela Gonzalez-Hernandez

**Affiliations:** 1 Department of Health Sciences, University of York, York, United Kingdom; 2 Department of Biostatistics and Epidemiology, Perelman School of Medicine, University of Pennsylvania, Philadelphia; 3 Center for Clinical Epidemiology and Biostatistics, Perelman School of Medicine, University of Pennsylvania, Philadelphia

## Abstract

**Importance:**

Statins are prescribed to help lower cholesterol levels but have poor adherence rates. Attitudes or beliefs toward medications are important to ascertain because they may be associated with patient behavior.

**Objective:**

To identify health-related discussion in Twitter posts mentioning a statin and analyze the content within these posts.

**Design, Setting, and Participants:**

This qualitative study included 11 852 posts related to 1 of 8 statins (7 licensed for use in the United States and 5 licensed in the United Kingdom) collected from May 10, 2013, to August 28, 2018. Twitter posts were identified as health related or not, and if health related, whether they were posted by a statin user, someone who knows a statin user, a health care professional, or undetermined. The health-related tweets were classified by the type of information posted, such as a belief about the medication, an adverse event, a question, or a reference to the cost of the medication. Data were analyzed from January 22 to November 19, 2019.

**Main Outcomes and Measures:**

The number of posts by each user type identified and the categories identified by analyzing the content.

**Results:**

Of the 11 852 Twitter posts about statins, 5201 (43.9%) were health related. The most frequent posts provided resource information, such as a link to a journal article (1824 of 5201 [35.1%]). The second most frequent type of health-related posts included personal beliefs or attitudes toward statins (1097 of 5201 [21.1%]). Personal experiences of adverse events (353 of 5201 [6.8%]), discussions about dosage (320 of 5201 [6.2%]), and questions about statins (191 of 5201 [3.7%]) followed. Posts indicated polarized beliefs and attitudes to statins from saving lives to causing death. Some beliefs, such as the direct confirmation that the use of statins mitigates the effects of an unhealthy diet, have not been extensively highlighted as common practice in the literature.

**Conclusion and Relevance:**

This qualitative content analysis of Twitter posts about statins provides insights into beliefs about statins. Patient perspectives gathered from social media may help to inform research and improve public health messages and communication between health care professionals and patients.

## Key Points

**Question:**

Can data from Twitter provide useful insights into patient beliefs and attitudes about statins?

**Findings:**

This qualitative study of 11 852 posts from Twitter containing a mention of a statin were manually categorized. In 5201 health-related posts, it was often possible to determine who was posting (eg, patient or health care professional) as well as derive content-relevant information (eg, beliefs and attitudes, adherence, adverse events, and cost).

**Meaning:**

Twitter may be used as a data source to find beliefs and attitudes that may affect patients’ decisions regarding their statin treatment.

## Teaser

This qualitative study identifies health-related discussion in Twitter posts mentioning a statin and analyzes the content within these posts.

## Introduction

Statins are one of the most frequently prescribed, cost-effective agents to decrease cholesterol levels.^1^ In the United States, atorvastatin calcium and simvastatin are the second and eighth most common outpatient prescriptions, respectively.^2^ Although statins reduce the risk of cardiovascular events, adherence to treatment is poor.^3,4^ Approximately one-third of nonelderly adults receiving statins are nonadherent,^4^ and interventions to improve medication adherence are largely ineffective.^5^ The reasons people stop taking statins are poorly understood.^3^

Guidelines on cardiovascular disease prevention recommend lifestyle changes alone or in addition to statins.^6,7^ Determinants of patient behavior, including medication adherence and lifestyle, are related to patient experience and attitudes and opinions about the medication.^3,8,9,10^ These determinants include adverse reactions,^11^ cost, and beliefs.^12,13,14,15^ Negative beliefs about a medication (such as fear of dependence) pose a stronger deterrent to adherence than other barriers, such as costs,^16^ and attempting to reduce copayments for cardiovascular drugs is largely ineffective.^17^

Traditional research on beliefs and attitudes relies on surveys,^18,19^ interviews, or focus groups.^9^ However, social media may allow the patient voice to be heard^20^ as well as provide additional data from a wider patient perspective and include patients reluctant to participate in research. In addition, social media posts can be collected in almost real time and are spontaneous; thus, they may be more likely to reflect true beliefs than research, which relies on interrogation. Social media can provide patients’ perspectives on conditions^21,22,23,24^ and adverse events^25^ but may also provide useful insight into patient attitudes toward medications. We categorized all Twitter posts (tweets) related to statins to ascertain the types of information posted, with particular attention to attitudes toward and beliefs about statins.

## Methods

Although many facets in reporting guidelines are outside the scope of this report, we adhere to relevant aspects of Consolidated Criteria for Reporting Qualitative Research (COREQ) reporting guideline.^26^ We identified posts on Twitter that mentioned a statin. Twitter is a convenient platform to use because of the public availability of posts and the relative ease of collecting massive amounts of data.^27^ Twitter is the third most popular social media platform in the United Kingdom^28^ and is used by one-quarter of people in the United States.^29^ All data used in this study were collected according to the Twitter terms of use and were publicly available at the time of collection and analysis. The protocol for this study was reviewed by the University of Pennsylvania’s institutional review board and was determined to meet the criteria for exempt human subjects’ research.

The tweets were collected with the Twitter application program interface stream using medication names and their variants.^30,31,32,33^ Collected Tweets were posted from May 10, 2013, to August 28, 2018, were in English, and contained a mention of a statin. Duplicate tweets, tweets with identical text but different tweet identifications, and retweets were removed. We searched for 8 statin medications, including atorvastatin, rosuvastatin calcium, pitavastatin calcium, simvastatin, pravastatin sodium, lovastatin, fluvastatin sodium, and cerivastatin sodium (Table 1). Seven were licensed in the United States (cerivastatin has been withdrawn) and 5 (atorvastatin, rosuvastatin, simvastatin, pravastatin, and fluvastatin) in the United Kingdom.^34^ Prescription rates show that atorvastatin and simvastatin dominate in both countries (Table 1).

**Table 1.** Statin Mentions in This Study and Prescription Rates in the United States and United Kingdom

Statin generic name (trade or brand name)	Mentions in tweets after duplication removal, No. (%)^a^	Total US prescriptions in 2016 in millions, No. (%)^b^	Total UK items prescribed in millions, No. (%)^c^
Atorvastatin calcium (Lipitor)	15 567 (60.1)	97 (44.5)	174 (50.7)
Rosuvastatin calcium (Crestor)	8996 (34.7)	20 (9.2)	10 (3.0)
Pitavastatin calcium (Livalo)	607 (2.3)	NR	0
Simvastatin (Zocor)	488 (1.9)	65 (29.9)	145 (42.1)
Pravastatin sodium (Pravachol, Pravigard pac)	119 (0.5)	25 (11.3)	144 (3.9)
Lovastatin (Mevacor, Altoprev, and Altocor)	72 (0.3)	11 (5.1)	0
Fluvastatin sodium (Lescol)	45 (0.2)	NR	0.6 (0.2)
Cerivastatin sodium (Baycol)	24 (0.1)	NR	0

Abbreviation: NR, not reported.

^a^ Includes 25 918 mentions.

^b^ Data are from ClinCalc DrugStats Database.^2^

^c^ Data are from January 8, 2014, to January 7, 2019 from OpenPrescribing.net.^34^

Data were analyzed from January 22 to November 19, 2019. This content analysis of tweets used a grounded theory framework^35^ in which themes were allowed to emerge from the data. No predefined set of criteria were used other than *drug or nondrug* and *health related or non–health related*. Classification criteria were developed after a first-pass annotation of the set of tweets. Annotation guidelines were devised and tested by 2 university researchers with experience in social media annotation (S.G. and K.O.) by individually annotating a sample of 500 tweets in Excel (Microsoft Corporation). Disagreements were resolved by consensus, and revisions were made to the guidelines. On the second round of annotations of 306 tweets, interannotator agreement measured using the Cohen κ^36^ was adequate at κ = 0.700, so no further revisions to the guidelines were required.

A last set of 1675 tweets was double annotated for interannotator agreement measurement. The interannotator agreement of these annotations was κ = 0.735, indicating substantial agreement.^37^

We were primarily interested in those categories of posts that could be informative to health-related research but also categorized irrelevant posts by the type of information posted. We then conducted a descriptive analysis of the tweets in each category. We did not collect demographic data on our sample.

## Results

We manually annotated 12 649 unique tweets (from 16 338) by 9116 users. We excluded 748 posts not related to statins (owing to typographic errors, spelling mistakes, or another use of the statin name), 31 computer-generated posts (such as bots), and 18 in a non-English language. This process left 11 852 posts for analysis, of which 5201 (43.9%) were health-relevant mentions and 6651 (56.1%) were not (Table 2). Non–health-relevant mentions tended to be jokes, financial market information, or online advertisements (Table 3). Most tweets were noncommercial in nature, and the 522 advertisements tended to present direct links to purchase statins online. The most frequent health-related posts provided factual information, such as a link to a journal article (1824 of 5201 [35.1%]).

**Table 2.** Categories of Health-Related Tweets Mentioning a Statin

Person tweeting	No. (%) of tweets (n = 5201)	Type of information about statins	No. (%) of tweets by Twitter user category^a^
Statin user discussing a drug they are taking, have taken, or will take and includes the expression of needing the medication	1707 (32.8)	Adverse event experienced	268 (15.7)
		Dosage	220 (12.9)
		Belief or personal opinions	145 (8.4)
		Cost to the user	139 (8.1)
		Efficacy	67 (3.9)
		Nonadherence (stopping or not taking as prescribed)	66 (3.9)
		Declined treatment	53 (3.1)
		Questions	45 (2.6)
		No further information disclosed	849 (49.7)
Someone known to the statin user discussing the medication/drug use of other people, such as a family member, friend, or colleague	346 (6.7)	Adverse event experienced	74 (21.4)
		Beliefs or personal opinions	38 (11.0)
		Dosage	28 (8.1)
		Cost to the user	17 (4.9)
		Questions	14 (4.0)
		Nonadherence (stopping or not taking as prescribed)	12 (3.5)
		Efficacy	11 (3.2)
		Declined treatment	2 (0.6)
		No further information disclosed	169 (48.8)
Health care professional discussing the medication, including the use of such by their patients	325 (6.2)	Dosage	70 (21.5)
		Cost to the user	26 (8.0)
		Questions	26 (8.0)
		Resource information	13 (4.0)
		Adverse event experienced	11 (3.4)
		Nonadherence	9 (2.8)
		Beliefs about medication	7 (2.2)
		Efficacy	6 (1.8)
		Declined treatment	1 (0.3)
		No further information disclosed	179 (55.1)
Inconclusive; not enough evidence to determine who is sending out the tweet but it is a health-related discussion	2823 (54.3)	Resource information	1811 (64.2)
		Beliefs or personal opinions	907 (32.1)
		Questions	106 (3.8)
		Dosage	2 (0.1)

^a^ Tweets could be coded with multiple subcategories; therefore, totals may be greater than 100%.

**Table 3.** Example Tweets by Category, Paraphrased to Maintain Anonymity

Statin-related tweet	Code definition	Example
Twitter user		
Statin user	First-person accounts of taking statin	I often told bar staff that I can’t have grapefruit because I’m on Lipitor.
Someone known to the statin user	Mentions use of statin by other people (eg, friend, family member, coworker)	My father died of an overdose of Crestor at age 82. His cardiologist LOVED the numbers, but didn’t bother to look at his patient, who had contracted rhabdomyolysis.
Health care professional	Identifies themselves as health care professional directly or through inference	I just prescribed my patient 40 mg Lipitor, 40 mg Lovenox, and 40 mg Lasix.
Inconclusive	Not enough evidence to determine who is sending, but it is a health-related discussion	Scientists Find New Tricks For Old Drugs (like Lipitor): https://www.nhpr.org/post/scientists-find-new-tricks-old-drugs#stream/0 and https://twitter.com/nhpr/status/1016482155168493568
		If you take Lipitor you should stop eating grapefruit.
Health-related information posted		
Adverse event experienced	Tweets state adverse drug reactions experienced or attributed to the intake of the drug	Good things going on so far in 2017...switched meds to tryglycerides and I can walk again! Apparently Atorvastatin is notorious for muscle stiffness.
Beliefs or personal opinions about medication	Tweets state a belief or a personal opinion about the medication or medication class	My dad says if I take my Lipitor I can eat as much bacon and cookies as I want to. He is a cardiologist so I figure he should know best.
		It is SuperBowl which means it’s time for the Baconator! 2 Lbs of bacon and 1Lb of sausage rolled together and cooked for 2 h into juicy goodness. I’ll take 2 Lipitors this evening.
		You should do research [on] Lipitor. It “eats” cholesterol. Your brain represents a quarter of your entire body’s cholesterol. Please research statins so you’ll STOP taking them to avoid the risk of you suffering from dementia later.
		I don’t care what the alt med says, no amount of diet and exercise is going to replace Lipitor.
		Lipitor is slow acting poison. A good diet and regular exercise will do a better job than that XXXX.
Cost to the user	Discussion about the cost/price of the medication they are taking	That is not totally accurate. I am on Crestor which is now a generic Rovastatin and I paid $16.00 yesterday with my insurance.
Declined treatment	Statements of declining the treatment	Why is that? I’m 54 and my cholesterol at my last physical was high according to my Doc. She recommended I take Lipitor. I said no thank you.
		I’ve been prescribed Lipitor but I declined to pick it up from the pharmacy. There’s no way in hell I’d take a statin.
Dosage	State the dosage of the medication	I’m on 5 mg of rosuvastatin now but I was on 80 mg of atorvastatin.
Efficacy	Tweet states the efficacy of the medication	Rosuvastatin is good stuff. LDL went from 165 to 43 in 3 mo on 20 mg.
Nonadherence (stopping or not taking as prescribed)	Tweets state the medication is not taken or is not taken as prescribed	I mistakenly took 2 Lipitor instead of 1 this morning, so I just ate twice as much fat to soak it up.
		Reading that just reminded me that I haven’t taken my Lipitor in 2 d.
		Lipitor gives me leg cramps so I break it in half. I don’t take a whole dose.
Questions, support, or advice about the medication/drug	The user is posing questions or is seeking information/advice about the drug, or options for disease management	Is anyone taking Crestor for high cholesterol? I was prescribed (not by my regular Doc) Lipitor for the first time, and I was hurting so bad just after 3 once-a-day pills. All my organs and muscles hurt so bad.
		Do you take the Lipitor before or after food?
		Is atorvastatin a preferred prescription in the NHS? I have a patient who goes back and forth to the UK and wants to start a medication he can continue there. Thanks.
Resource information	Tweet gives information about the drug/medication, includes research and articles	Atorvastatin attenuates the ovarian damage induced by cyclophosphamide in rat: An experimental study. https://pubmed.ncbi.nlm.nih.gov/30027148/?dopt=Abstract&utm_source=dlvr.it&utm_medium=twitter
		#HealthTip: Know your risks and benefits: For every 255 people who take the cholesterol-lowering medications called statins (eg, Lipitor, Crestor), 5.4 people are saved from a stroke or heart attack while 1 person develops diabetes as a side effect.
No information disclosed for categorization	NA	I am now on statins.
		I can’t because grapefruit interacts with my Lipitor.
Non–health-related mentions		
General mentions of the drug	Tweet refers to the drug but not related to a discussion directly about health. Some examples of these types include cultural references, jokes, financial or news reports, and online pharmacies	I don’t like that Crestor commercial. She clearly doesn't diet or exercise or she wouldn't be overweight like that.
		Global Rosuvastatin Market 2018 Current and Future Prospect: Cadila Pharmaceuticals, AstraZeneca, MSN Laboratories and Teva Pharmaceutical Industries.

Abbreviations: LDL, low-density lipoprotein; NA, not applicable; NHS, National Health Service.

**Figure 1.**
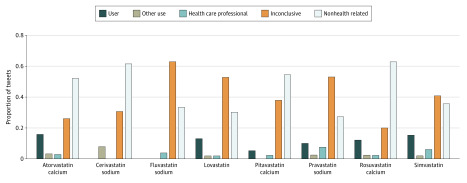
Distribution of Health-Related and Non–Health-Related Tweets by Statin Proportions of tweets are given as those posted by statin users (user), those posted about someone they knew taking statins (other’s use), health care professionals, inconclusive identification (inconclusive), and non–health related.

Figure 1 shows that each type of statin had a different pattern of mentions. Although those most commonly prescribed (Table 1) were mentioned more by users, others, such as fluvastatin, are mentioned more in an informational context. Figure 1 also demonstrates the effect of general public discourse on the distribution within our set. Rosuvastatin was the fourth most prescribed statin in the United States and United Kingdom (Table 1) but was the second most mentioned in our data set. Most of these tweets were non–health related, dominated by comments about television commercials and the US President’s use reported in his publicly released physical examination results.

### Health-Related Mentions

#### Person Posting

For 1707 of 5201 tweets (32.8%), we could ascertain that the post was from a statin user (Table 2). These posts often used the pronoun *I* or used *my* followed by the statin name. We identified 346 of 5201 tweets (6.7%) as from people posting about someone they knew taking statins, such as a parent or a partner (Table 2). A further 325 posts (6.2%) were from health care professionals (Table 2), sometimes a physician or pharmacist, although in most cases it was not possible to decipher the profession. These posts often referred to a *patient* or *pt*, but in all cases the patient was unnamed and untraceable.

For some posts by users, someone known to the user, or health care professionals, no further information could be extracted from the post (Table 2). With the other health-related mentions (2823 of 5201 [54.3%]), we were unable to determine the type of person posting from the tweet itself. These posts tended to provide a resource, such as sharing journal articles or factual information (1811 of 2823 [64.2%]) (Table 2).

**Figure 2.**
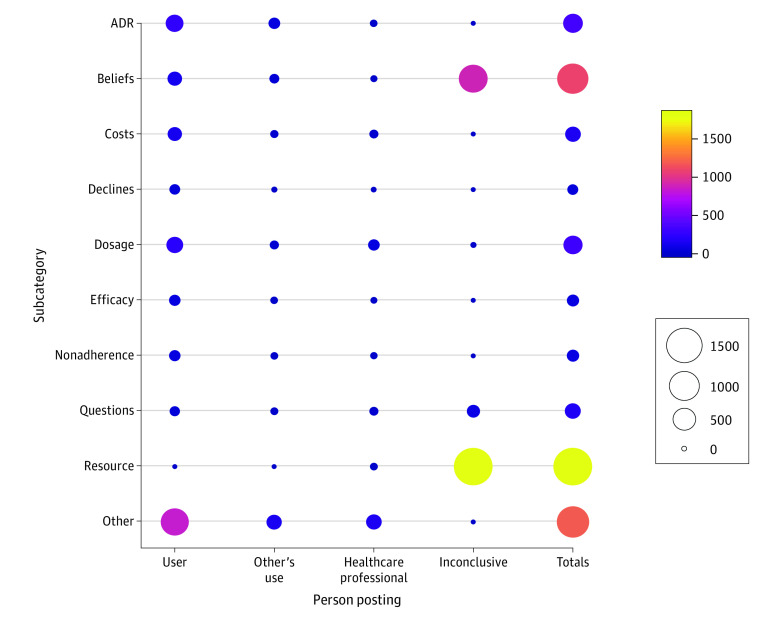
Health-Related Tweet Subcategories About Statins by Person Posting Persons posting tweets include those posted by statin users (user), those posted about someone they knew taking statins (other’s use), health care professionals, and inconclusive identification (inconclusive). ADR indicates adverse drug reaction. Values in the color bar and circle sizes indicate numbers of persons posting.

Figure 2 demonstrates the dominance of resource of information and beliefs by inconclusive users and no further information posted by statin users. Although the most common types of posts may not be the most informative, the numbers in the other categories are such that we are still able to uncover potentially useful information.

#### Beliefs About Medication 

Of 1097 posts (21.1%) concerning personal beliefs or attitudes about statins, 787 (71.7%) referred to risk compensation behaviors in which patients engage in behaviors such as poor diet and physical inactivity while perceiving themselves to be protected or at lower risk by virtue of taking preventative medications.^38^ Most tweets (666 of 1097 [60.7%]) did so without clarity of whether the behavior was acted on. Others (121 of 1097 [11.0%]) were in relation to the user’s own behavior, such as the freedom to eat an unhealthy diet (eg, “with Lipitor I eat as much bacon and cookies as I want”) or increasing their dose of statins after eating unhealthy food.

Some users posted about the beneficial effects of statins (51 of 1097 [4.6%]). These referred to the lowering of cholesterol levels, cleansing of the arteries, and prevention of cardiovascular events and death or were more generic about statins being a “magic” or “proven 98% effective” drug.

Harm or medical mistrust accounted for 219 of 1097 posts about beliefs (20.0%). This was either a general reference to statins as “dangerous” or “poisonous,” with named adverse events, such as dementia, liver failure, and mortality (eg, “Lipitor kills ur liver”), or a reference to profits driving false claims of benefit from a corrupt drug industry or government. Others discussed alternative strategies to statins, such as their preference to lower cholesterol with lifestyle changes or their preference for statins rather than alternative therapies (41 to 1097 [3.7%]).

#### Adverse Events 

A total of 353 posts (6.8%) concerned personal experience of adverse events. Many of these tweets referred to muscle pain or cognitive issues. Others made less specific references to unnamed “side effects” or statins “killing people.” Such events often led to posts about switching medications or stopping taking statin therapy (eg, “I was on Lipitor, switched to generic when the pain started”).

Adverse events were mentioned most commonly by the individual experiencing them (268 of 353 [75.9%]). However, people also posted about family members, typically a partner or a parent, and occasionally friends (74 of 353 [21.0%]).

#### Dosage 

A total of 320 health-related posts (6.2%) discussed drug dosage. These posts were generally neutral and were related to changes in dosage or dosages available or tolerable. Posts often gave numerical data (eg, “I was on 80 mg Lipitor … now I’m down to 40 mg. Yippee.”).

#### Questions About Medication 

A total of 191 health-related posts (3.7%) included questions about statins. These questions were wide ranging, including adverse effects, drug interactions, dosages, or practicalities. Questions from health care professionals (26 of 191 [13.6%]) often contained relatively detailed circumstances regarding a specific patient.

#### Cost 

One hundred eighty-two health-related posts concerned costs (3.5%). Among the discussions on personal costs, many were related to whether statins were covered by health insurance policies, how much generic versions of the drug cost, comparisons of different suppliers of the drugs, and comparisons of the cost in different countries.

#### Nonadherence

Eighty-seven health-related posts (1.7%) discussed nonadherence. The largest type of nonadherence discussed was nonconforming (42 of 87 [48.3%]), meaning the patient does not take the medication as prescribed,^39^ such as missing dose(s) (25 of 42), taking extra dose(s) (11 of 42), or taking a smaller dose (2 of 42). Many missed doses were due to forgetting, not refilling the prescription in time, or delays by the prescriber. Reasons for taking an extra dose were not often explained but sometimes were to make up for poor dietary choices. Those who took a smaller dose did so to try to mitigate adverse reactions.

Nonpersistence, in which the patient decides not to continue with treatment, was the next largest type of nonadherence discussed (40 of 87 [46.0%]). Some users discontinued without giving a reason (7 of 40); however, others stopped owing to adverse reactions (13 of 40); controlling their cholesterol level with diet, exercise, or supplements (11 of 40); or affordability (2 of 40). Others discontinued owing to beliefs that the statin was bad or poison or that they were now cured because their blood levels were normal (7 of 40).

Primary nonadherence, in which the patient never filled the prescription for the statin, was discussed in 5 of 87 posts (5.7%), with reasons stated being the fear of adverse reactions, medical mistrust, or the belief that altering the diet was a better option. Some posts were not categorized as nonadherent when they may have belonged in this category, because it was difficult to decipher whether stopping use or changing the dosage was directed by a health care professional.

#### Efficacy 

Eighty-four health-related posts (1.6%) concerned efficacy. These posts differed from the positive-belief posts because they were a statement of fact on effectiveness. These posts commonly demonstrated efficacy with a reduction in cholesterol levels, often supported with numerical data.

#### Declining Statin Therapy 

Fifty-six posts (1.1%) concerned declining statin therapy. Refusal often stemmed from concerns regarding the adverse effects of statins. Others believed that they would like to try alternative strategies, such as changes to their diet (eg, “My ‘white coat’ wants me to start on Lipitor because my cholesterol is a little high. I said no thanks, I am gonna change my diet. He says ‘that won't make any difference.’”).

## Discussion

This research demonstrates how social media content may elucidate attitudes and beliefs toward a medication. Although content analyses of social media have been conducted to uncover beliefs and attitudes about specific diseases or conditions,^21,22,23,24^ it has rarely been used to uncover attitudes about medications beyond specific questions, such as the patient’s preferred drugs,^40^ or quality of life^41^.

Patient perspectives of statins have previously been assessed using qualitative studies, such as surveys, interviews, and focus groups.^9,18,19^ This research showed that these studies could be further enriched or informed through analysis of social media data. A systematic review including 888 participants^9^ and a survey of 10 138 statin users^18^ did not identify the “license to be unhealthy” belief identified herein, and although medical mistrust was identified,^9,18^ this was only related to mistrust in health care professionals. Through social media, we identified distrust in pharmaceutical companies and governments.

The literature has overlapping themes with the present study, such as discussions on the benefits, adverse effects, and cost.^9,18^ However, some issues were uncovered in more depth in the literature, such as “taking control” and “routinizing into daily life.” This may reflect the lack of depth that can be obtained through social media research.

Several studies have also examined the effect of statin use on dietary changes with contradictory results. Although some studies found that statin users had an increase of fat and caloric intake and a faster increase in body mass index compared with nonstatin users,^42^ others have found no change^43,44^ or a decrease.^45,46,47^ In this study, however, Tweets suggested a potential increase in fat and caloric intake with those taking statins and a healthier diet in those choosing changes in lifestyle in place of statins. Concerns were identified in the tweets that drug manufacturers are being dishonest and that statins are “poisonous” or at least do more harm than good. Sharing of incorrect or exaggerated information on Twitter has been identified in previous research.^48,49^ Indeed, social media has been found to identify extremes in views not always uncovered via traditional surveys. Trends in information being exchanged in social media may be important, because studies have identified that events such as news stories can increase or decrease the risk of early discontinuation of a drug.^50,51^

More than half of the tweets on statins (56.1%) were not health related. This finding is in agreement with other research^27^ that 41% to 66% of posts on diseases and conditions are nonmedical.^27^ We identified that 353 posts with a statin mention contain an adverse event report (3.0% of 11 852), which is similar to rates in the literature ranging from 0.2% to 8.0% of posts.^25^

Our research demonstrates that although many posts on Twitter are of a trivial nature,^52^ with such a vast source of data—even if most tweets are not relevant to the research question—substantial numbers of relevant posts can potentially be identified. Effective communication strategies are paramount to implement shared decision-making based on informed risks and benefits. This is particularly the case for preventative medications, such as statins, with which patients may experience adverse effects and the numbers needed to treat to prevent a cardiovascular event may seem high to patients.^53^ Social media may help inform such communication strategies and public health messages by making popular beliefs and attitudes well known. Beliefs and attitudes may be specific to particular drugs. Messages can be aligned to dispel any generally held concerns or misconceptions and can help to inform conversations on issues that may be of particular importance to the patient, such as diet, adverse events, and cost.

### Strengths and Limitations

Research methods used in this study were straightforward and quick compared with traditional qualitative research, although manual rather than automated annotation increased the length of time taken to annotate. We also captured routine populations who may not be represented in randomized clinical trials or traditional research designs, and we were able to eliminate reporting bias that occurs from speaking with a researcher.

However, this study also has some limitations. Although we searched for multiple terms for the individual statins, we did not include general terms, such as *cholesterol medicine*. A future study could expand on the terms used. We were also limited to publicly available Twitter accounts and the collection limits enforced by Twitter.

We did not extract demographic data (we would have had to refer to users’ profiles or further posts in their timelines), and sometimes we were unable to understand the post without its context. We did not study cultural differences. For example, US citizens may be more likely to mention cost than people from the United Kingdom. In addition, some cultures may have different stopping rules for statins.

The generalizability of our findings is another limitation. People who post about statins on social media may not be representative of the population of statin users, and people will be selective on what they choose to post. It is already known that social media users tend to be younger^54,55,56^ and that statins are used in an older population. The median age of a US Twitter user is 40 years vs the median age of a US adult, which is 47 years.^56^ Twitter users also tend to have higher incomes and attain a higher level of education.^56^ A large survey identified that former statin users are more likely to rely on the internet as a data source than current statin users.^18^ However, studies have found that in some respects, social media users tend to reflect the distribution of the population, for example, in respect to sex^57^ and race or ethnicity.^56^

We also limited our research to 1 social media platform and 1 drug category. Statins are commonly used and mentioned on social media; however, the amount and type of comments may not be typical of all drug classes.

## Conclusions

This qualitative study identified interesting beliefs and opinions regarding statins that may affect patient behavior. Social media may be useful for investigating public prevailing attitudes when investigating particular medications as well as patient-reported adverse events and issues relating to accessibility (such as cost). The approach to systematic analysis of social media data should be generalizable to other medications, with perhaps some variation in the specific subtopics, whereas the specific content-related findings might be exclusive to this drug class.

Public perceptions about medications could help inform research, particularly when developing research questions or recruitment and implementation strategies. Specific to statins, public perceptions elucidated by this study could be used to help inform and improve public health messages and communication between health care professionals and patients.
